# Asymmetric affective polarization regarding COVID-19 vaccination in six European countries

**DOI:** 10.1038/s41598-024-66756-w

**Published:** 2024-07-10

**Authors:** Maximilian Filsinger, Markus Freitag

**Affiliations:** 1 Institute of Political Science, University of Bern, Fabrikstrasse 8, 3012 Bern, Switzerland; 2 Multidisciplinary Center for Infectious Diseases (MCID), University of Bern, Hallerstrasse 6, 3012 Bern, Switzerland

**Keywords:** Human behaviour, Epidemiology

## Abstract

While recent research has shown that supporters and opponents of COVID-19 vaccination have polarizing political attitudes and beliefs, we lack a thorough understanding of how these two groups think about each other. To investigate the feelings and stereotypes between supporters and opponents of COVID-19 vaccination, this study draws on cross-sectional survey data from six European countries (France, Germany, Italy, Spain, Switzerland, and the United Kingdom), collected between January and March 2022 (n = 6379). Our findings indicate an opinion-based affective polarization between supporters and opponents of COVID-19 vaccination. Both groups not only adopt different positions on the issue but also display dynamics of in-group favoritism and out-group hostility. Most notably, our assessment of thermometer scores and character trait ratings shows that this affective polarization is asymmetric, as it is stronger among the pro-vaccination group. Our findings are critical to the control of infectious diseases because affective polarization has been shown to influence health behaviors such as compliance with government policies. The issue is even more pressing as globalization boosts the threat of pandemic emergence and accelerates the global transmission of diseases.

## Introduction

The advent of COVID-19 vaccines was hailed by many as a promising path to global immunity and a return to pre-pandemic routines. However, the vaccination efforts have not unfolded without controversy. Initially seen as a unifying element against the disease, the vaccine has paradoxically become a source of contention that continues to divide societies even after the pandemic has lost its threat^1–3^. While a majority of people supported and received the vaccine, a consistent minority refused it^2,4,5^. These two groups differ significantly not only in their position on the vaccination, but also in other sociopolitical attitudes^2^. Vaccination status also appears to induce discriminatory behavior toward the respective out-group^1,6,7^. While research has revealed that in the context of COVID-19, the anti-vaccination and the pro-vaccination group express conflicting attitudes and beliefs, we know surprisingly little about how they feel about and perceive each other.

To fill this lacuna, we apply the concept of affective polarization, i.e., the emotional attachment to in-group members and hostility toward out-group members, to the issue of COVID-19 vaccination^8,9^. Polarization has traditionally been understood in ideological terms, implying a divergence of political preferences of citizens, political elites, or parties^10^. In recent years, scholars have fundamentally shifted their attention to affective polarization, which—although linked to ideological polarization—represents a distinct form of polarization that is partly rooted in social identities^8,11–14^.

In this report, we aim to expand the scope of possible social identities and argue that such polarization can also arise from identification with opinion-based groups^15–17^. Building on social identity theory, we hypothesize that major issues such as COVID-19 vaccination can generate affective polarization by prompting people to align themselves with others based on a shared opinion about the issue. Drawing on key insights from social identity theory, we argue that the mental processes of social categorization, social identification, and social comparison shape the social identities of both the anti-vaccination and the pro-vaccination group. However, we expect that the two groups’ judgements of the respective in-group and out-group will differ due to differences in the perceived salience of the respective out-group.

To assess opinion-based affective polarization between opponents and supporters of COVID-19 vaccination, we make use of an online survey conducted simultaneously in France, Germany, Italy, Spain, Switzerland, and the United Kingdom between January 25 and March 8, 2022 (n = 6379). The survey was administered by the company SurveyEngine using quota sampling for gender, age, and education (as well as language in the case of Switzerland) yielding around 1000 respondents per country (see supplementary material, section A, Tables 1–10). The timing of the survey is exceptional because the data was collected during intense public debates about the value and consequences of the COVID-19 vaccination in all six countries.

Our correlational analyses of two popular measures of affective polarization suggest that both groups are antagonistic toward each other. That is, COVID-19 vaccination supporters (opponents) view COVID-19 vaccination opponents (supporters) negatively and COVID-19 vaccination supporters (opponents) positively. It should be noted, however, that both in-group attachment and out-group dislike appear to be stronger among the pro-vaccination than among the anti-vaccination group, resulting in an asymmetric pattern of affective polarization.

This paper makes a threefold contribution. First, we advance a conceptualization of opinion-based affective polarization by focusing on COVID-19 vaccination as a major issue that has generated a global debate. Second, we employ two common approaches to measuring affective polarization—thermometer scores and character trait ratings—between the pro-vaccination and the anti-vaccination group, thereby shedding additional light on the conflict around COVID-19 vaccination. Third, our study contributes to our understanding of the social and political consequences of the COVID-19 pandemic, because numerous studies have shown that affective polarization destabilizes social cohesion and undermines democratic norms^9,18–22^. What is more, understanding vaccine-related divides is crucial for the development of effective public health policies, as affective polarization hinders their implementation^22,23^, thus undermining efforts to control the spread of infectious diseases and to limit their consequences for the health of the citizenry^24–26^. Consequently, understanding vaccine-related affective polarization is critical, as the consequences reach deep into the political and social fabric of democratic societies^27–29^. The issue is also pressing because globalization boosts the threat of pandemic emergence and accelerates global disease transmission^30^.

## Background

Affective polarization, understood as emotional attachment to in-group partisans and hostility toward out-group partisans, has become an important component of modern democracies^8,9^. Rooted in social identity theory^31^, the concept of affective polarization has long been studied with a focus on partisanship^8,32^. However, the identification and subsequent affective evaluation of in- and out-groups can also be based on identification with a common opinion rather than a party label ^2,15,33–36^. Particularly after dramatic events such as natural disasters, opinion-based affiliations tend to form around controversial issues that prompt diametrically opposed groups regarding the preservation of the status quo^36^. This is especially true for issues that cross-cut traditional lines of social categories or partisan conflict. More than just a tally of rational considerations^37^, this form of polarization rests on the assumption that people define their opinions as part of their identity, i.e., they “need to define themselves in terms of their opinion group membership in the same way that they would any other meaningful social group”^15^. Such opinion-based social identities contribute to the self-esteem of group members and thus inevitably lead to a tendency for positive in-group evaluation and a clear distinction from others outside the group^37^.

According to social identity theory, three mental processes are required for the formation of a social identity^31^. First, social categorization, in which we distinguish between “us” and “them”. Second, social identification, in which we adopt a social identity based on our group membership, implying a sense of shared identity and belonging. Third, the social comparison of our in-group with an out-group, which in the context of group competition generates not only positive feelings toward the in-group but also negative feelings toward the out-group and thus conflictive intergroup relations, which in turn reinforce in-group identification^31^.

**Figure 1:**
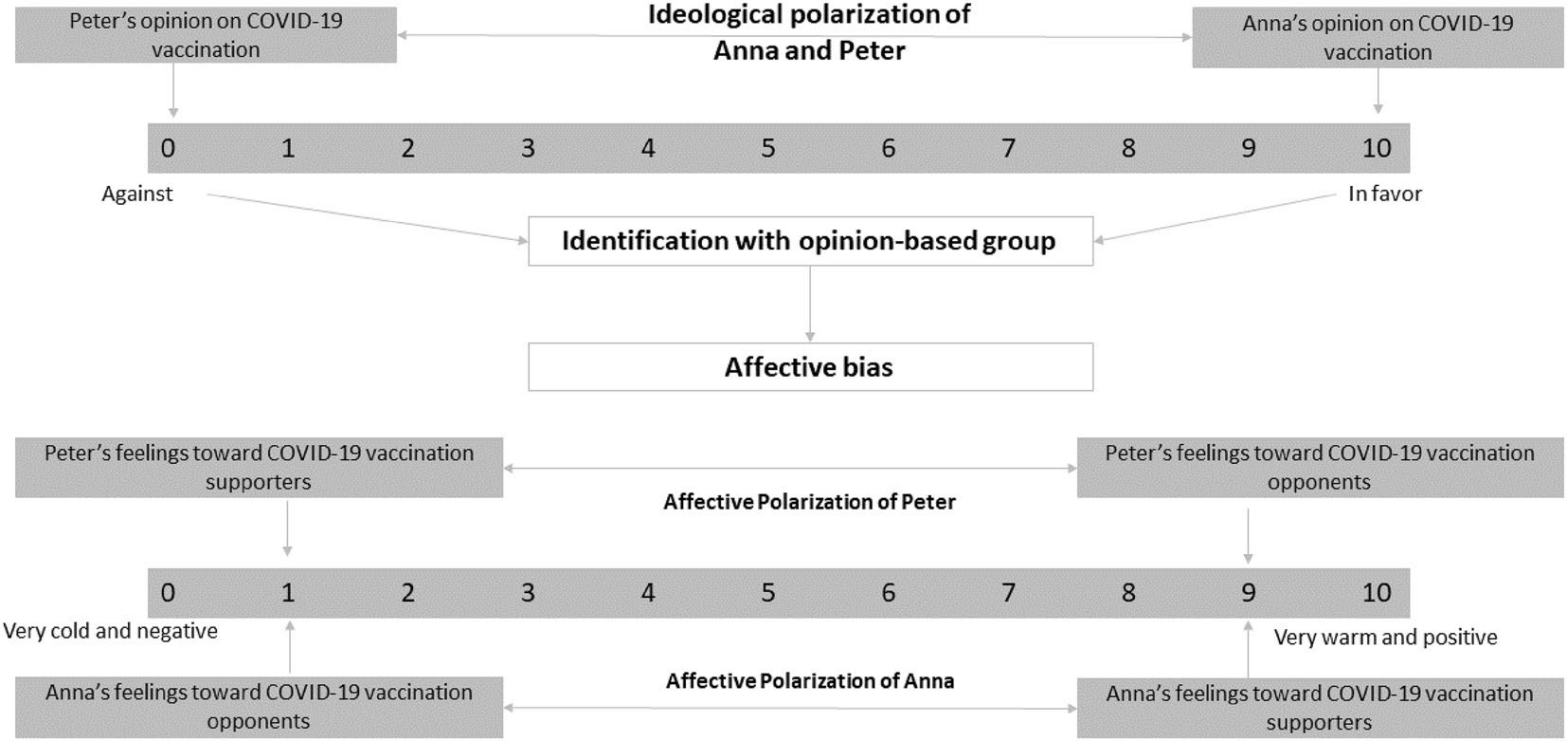
Psychological processes of ideological and affective polarization regarding COVID-19 vaccination based on social identity theory (conceptual model).

In this study, we focus on the formation of opinion-based affective polarization regarding COVID-19 vaccination. To illustrate this, Fig. 1 provides a conceptual model with two fictional characters, Anna and Peter, their opinions on COVID-19 vaccination, and their feelings toward supporters and opponents of this specific vaccine. While Peter is opposed to COVID-19 vaccination, Anna is in favor of it (ideological polarization, upper part of Fig. 1). Based on their respective ideological position with respect to the issue, Anna and Peter categorize themselves as pro-COVID-19 vaccine and anti-COVID-19 vaccine, respectively.

This categorization is followed by a process of identification in which both begin to develop an opinion-based identity with their respective group. In other words, they start to see themselves in terms of their group’s characteristics and incorporate its norms, values, and behaviors^31^. Thus, Anna sees herself as a member of the pro-COVID-19 vaccine camp and identifies with others from that camp, while Peter sees himself as a member of the anti-COVID-19 vaccine camp and connects with others from that camp (middle part of Fig. 1).

Having categorized and identified with one group, Anna and Peter begin to compare their group to the other. In a context of group competition, such comparisons are biased in favor of one’s in-group in order to maintain self-esteem, which often leads to negative feelings toward the out-group^16,17,31^. The end result therefore is an affective bias between the groups that stems from the mental processes of categorization, identification, and comparison^16^.

Based on these considerations, we expect opinion-based group formation and affective polarization regarding COVID-19 vaccination. The debate about the benefits of vaccination and vaccine mandates was highly politicized during the COVID-19 pandemic. Previous research also suggests that divisions based on vaccination status emerged during the pandemic^1–3^. Thus, we consider it likely that the issue of COVID-19 triggered opinion-based group identification, as it was highly salient at the time and prompted individuals to take sides during this watershed public health crisis^16^. In other words, we expect pro-vaccination individuals to identify with others who share their views and to feel positive about other pro-vaccination individuals and negative about anti-vaccination individuals, while we expect the opposite for anti-vaccination individuals. Thus, if our arguments are supported, our data would show that Anna, as a pro-COVID-19 vaccination individual, would feel positive toward other pro-COVID-19 vaccination individuals and negative toward anti-COVID-19 vaccination individuals. The opposite pattern should be observed for Peter (lower part of Fig. 1).

While the key insights of social identity theory make us suggest that both groups would express affective polarization in similar ways, the perceived salience of the out-group is crucial for this intergroup affect^31^. In this regard, recent research indicates that both groups will differ in their expressed levels of intergroup affect^1,2,16^. In our case, one might expect higher levels of intergroup affect among vaccination supporters because they view immunization as a social contract to achieve collective security^5,38^. Consequently, vaccination opponents break this contract and act as free riders and norm violators^5,38^. In this vein, one might assume high levels of affective polarization among vaccination supporters because the out-group (vaccination opponents) is considered a rule-breaker, norm violator, and potentially threatening.

Conversely, opponents of the vaccine may focus their antipathy not on supporters of the vaccine but on political actors, such as the government. In their view, the norm violators are government actors who promote a vaccine that is seemingly unnecessary given the supposed controllability of the pandemic. In some countries, anti-vaccination groups have frequently taken to the streets, sometimes violently, to voice their discontent with the restrictions on their individual freedom imposed by lockdowns or vaccination policies^39^. However, these protests were often directed against the government. The regression analyses in the supplementary material, section B (Table 11) show that in all six countries studied, anti-vaccination individuals were significantly more distrustful of the government than pro-vaccination individuals were. Consequently, the negative affect toward vaccination supporters could be lower. Thus, the difference in salience of the opposing group for pro- and anti-vaccination individuals might have important implications for the level of intergroup affect. As a result, one might expect both in-group attachment and out-group dislike to be higher among the pro-vaccination group than among the anti-vaccination group. Referring to our conceptual model, Peter should be less affectively polarized than Anna.

## Results

### A. Measuring opinion-based affective polarization regarding COVID-19 vaccination using thermometer scores

We assessed opinion-based affective polarization regarding COVID-19 vaccination in a multistep process. To begin with, we asked respondents to indicate on an 11-point scale ranging from 0 “complete rejection” to 10 “complete support” how strongly they oppose or support COVID-19 vaccination (Mean (M)_Sample_ FRA = 6.83, (M)_Sample_ GER = 7.8, (M)_Sample_ ITA = 7.7, (M)_Sample_ SPA = 7.96, (M)_Sample_ SWI = 7.26, (M)_Sample_ UK = 8.14).

**Figure 2:**
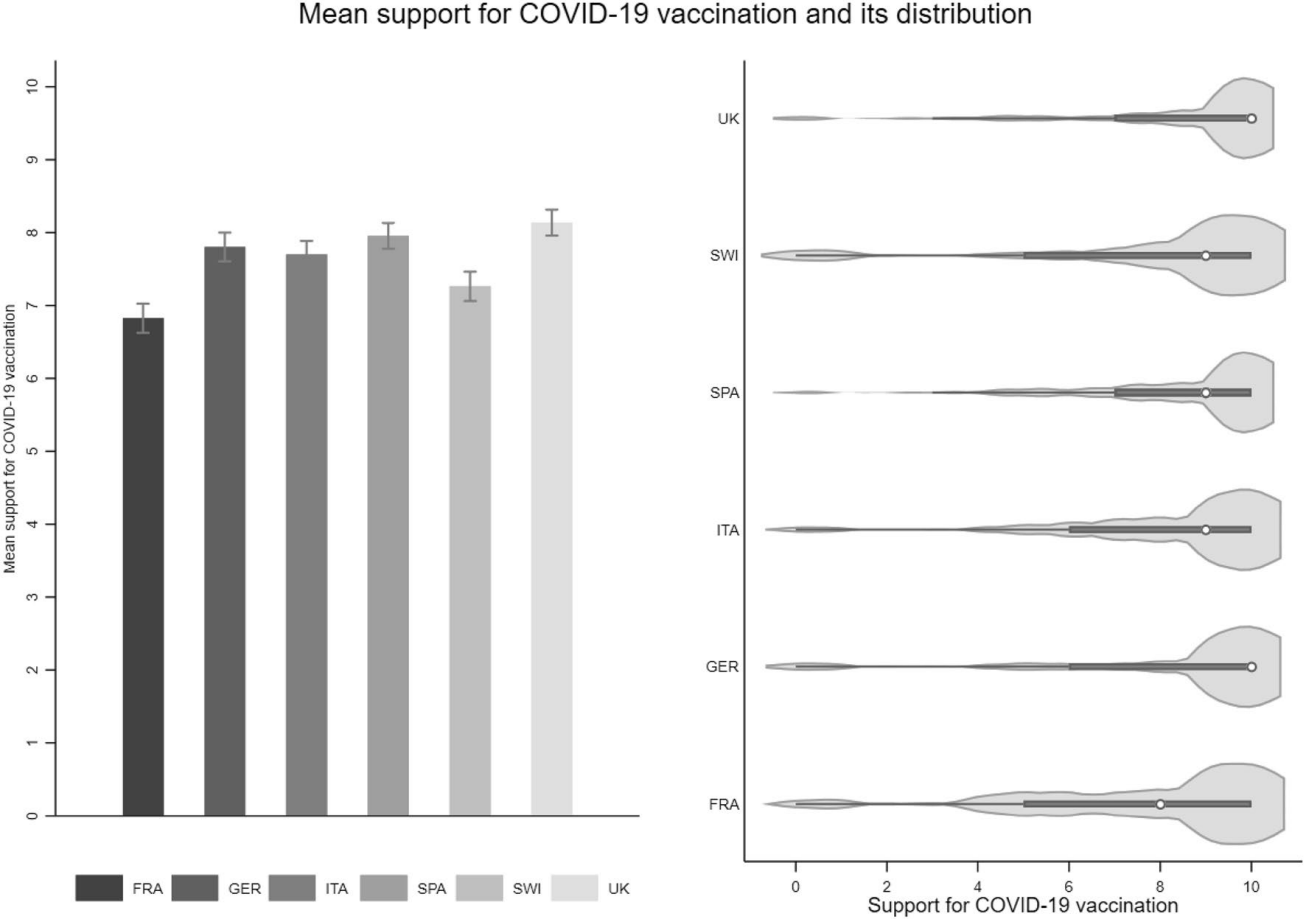
Mean support for COVID-19 vaccination and its distribution.

The left panel in Fig. 2 shows the mean levels of support for COVID-19 vaccination across our six countries. Generally, support is very high with mean levels of 7 on the scale from 0 to 10. The lowest level of support is found in France and the highest in the UK and Spain. The violin plots in the right panel of Fig. 1 again illustrate this finding as the distribution is skewed in favor of COVID-19 vaccination. However, the distribution shows that a consistent minority opposes COVID-19 vaccination in all six countries. That is, the issue has triggered the formation of two groups: one for and one against it.

In a next step, we applied the most widely used measurement approach for affective polarization, the feeling thermometer^8,40^, in which respondents are asked to rate their feelings about a specific subject on a temperature scale^9,40^. First, we dichotomized the variable on the position regarding COVID-19 vaccination and classified respondents who indicated a value from 0 to 4 as opponents of COVID-19 vaccination (anti-vaccination group) and those who indicated a value from 6 to 10 as supporters of COVID-19 vaccination (pro-vaccination group). We excluded respondents who indicated a value of 5 as they were neutral on the issue. Second, we asked respondents of the respective groups to rate their feelings toward a) supporters of COVID-19 vaccination and b) opponents of COVID-19 vaccination on a scale from − 5 (“very cold and negative”) to + 5 (“very warm and positive”). We transformed this scale to range from 0 to 10 and subsequently used the absolute difference between the two ratings to obtain a measure for affective polarization ((M)_Sample_ FRA = 5.1, (M)_Sample_ GER = 6.05, (M)_Sample_ ITA = 6.12, (M)_Sample_ SPA = 6.44, (M)_Sample_ SWI = 5.32, (M)_Sample_ UK = 6.03). The descriptive results are reported below using bar graphs for readability. Formal t-tests are reported in the supplementary material, section C, Tables 12–14.

**Figure 3:**
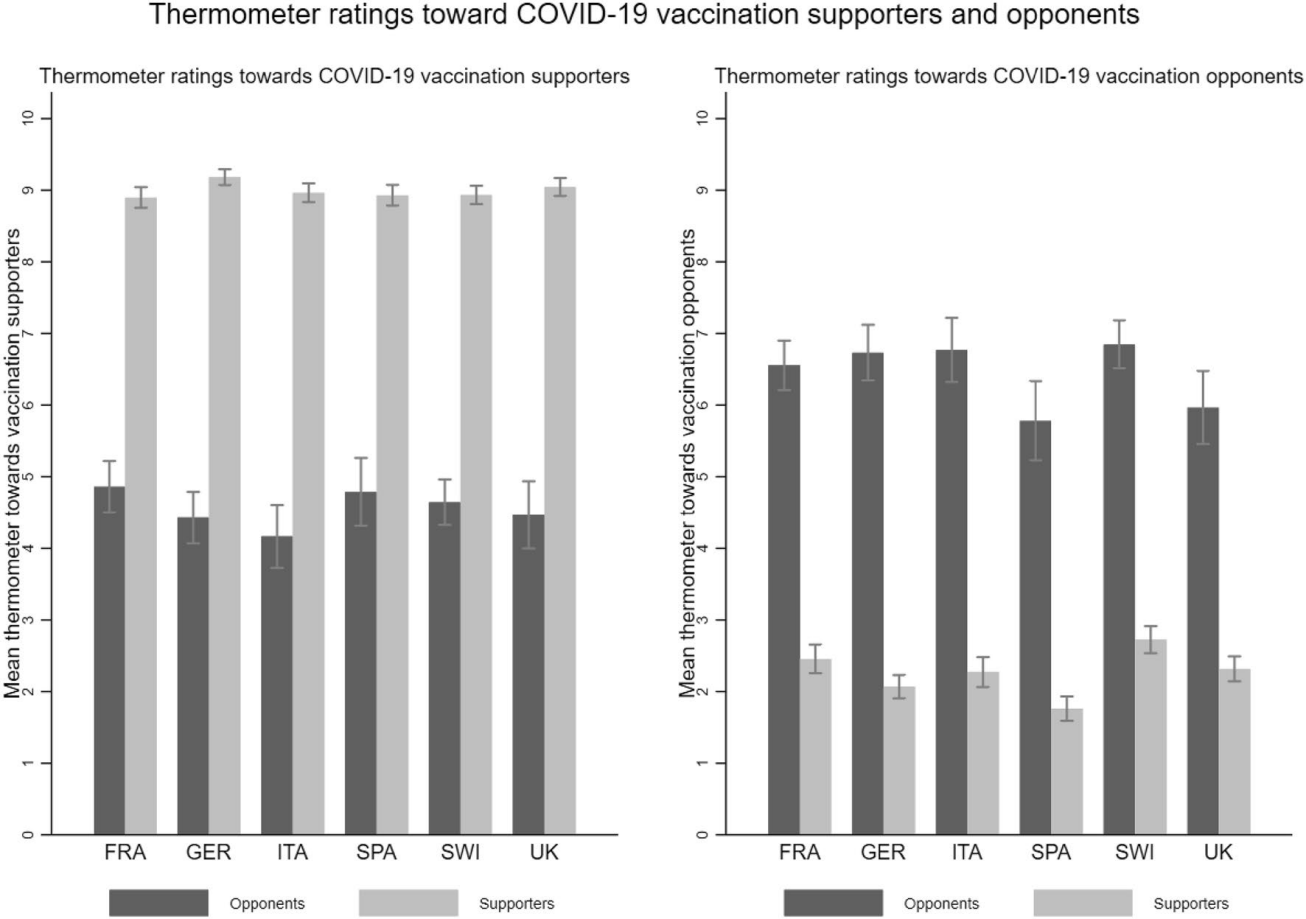
Thermometer ratings of feelings toward COVID-19 vaccination supporters and opponents by group and country. Notes: Figure 3 shows the mean thermometer ratings of feelings toward vaccination supporters and opponents separated by group and country, with 95% confidence intervals. Reading example for France in the left panel: In France, vaccination opponents have an average thermometer rating of feelings toward vaccination supporters of 6.15, while vaccination supporters have an average thermometer rating of feelings toward vaccination supporters of 7.04 on a scale of 0–10. The difference is statistically significant.

Figure 3 shows the thermometer scores for the two groups separated by group membership (supporters vs. opponents) and country. The feelings toward COVID-19 vaccination supporters (left panel in Fig. 3) show a clear pattern: Supporters feel very positive and warm toward other supporters ((M)_Supp_ FRA = 8.9, (M)_Supp_ GER = 9.18, (M)_Supp_ ITA = 8.96, (M)_Supp_ SPA = 8.93, (M)_Supp_ SWI = 8.93, (M)_Supp_ UK = 9.05). Conversely, opponents feel somewhat cold and negative toward supporters, with values below the neutral value of 5 ((M)_Opp_ FRA = 4.86, (M)_Opp_ GER = 4.43, (M)_Opp_ ITA = 4.17, (M)_Opp_ SPA = 4.79, (M)_Opp_ SWI = 4.65, (M)_Opp_ UK = 4.47). These values are statistically significant and different at the 95% level.

Looking at the thermometer scores for COVID-19 vaccination opponents, we see a mirror image. Opponents feel relatively positive and warm toward other opponents ((M)_Opp_ FRA = 6.55, (M)_Opp_ GER = 6.73, (M)_Opp_ ITA = 6.77, (M)_Opp_ SPA = 5.78, (M)_Opp_ SWI = 6.85, (M)_Opp_ UK = 5.96). Yet, these positive in-group feelings are comparatively lower among opponents than among supporters. It seems that there is less group cohesion among opponents than among supporters of COVID-19 vaccination. Furthermore, supporters express very cold and negative feelings toward opponents, as expected ((M)_Supp_ FRA = 2.46, (M)_Supp_ GER = 2.07, (M)_Supp_ ITA = 2.27, (M)_Supp_ SPA = 1.76, (M)_Supp_ SWI = 2.73, (M)_Supp_ UK = 2.32). All differences between supporters and opponents are statistically significant at the 95% level.

**Figure 4:**
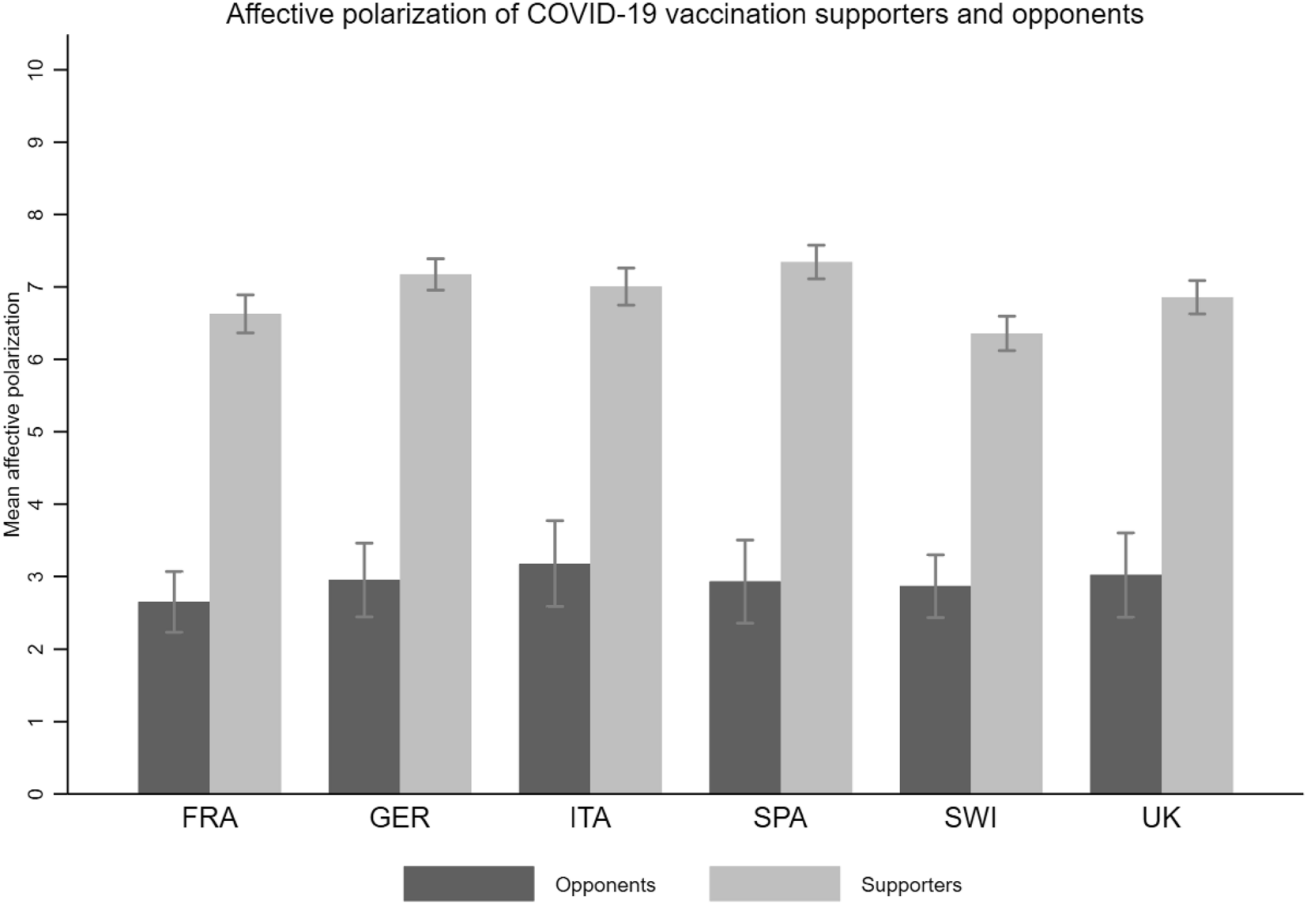
Affective polarization of COVID-19 vaccination supporters and opponents. Notes: Figure 4 shows the mean level of affective polarization (thermometer measure) by group and country, with 95% confidence intervals. Reading example: In France, vaccination opponents show an average affective polarization of 2.65 and vaccination supporters of 6.63 on a scale from 0 to 10. The difference is statistically significant.

Figure 4 reveals the absolute difference between both thermometer ratings, separated by group and country. Supporters show a relatively high average difference in feelings toward the in-group and the out-group ((M)_Supp_ FRA = 6.63, (M)_Supp_ GER = 7.17, (M)_Supp_ ITA = 7.01, (M)_Supp_ SPA = 7.35, (M)_Supp_ SWI = 6.46, (M)_Supp_ UK = 6.86). Opponents also show a difference in feelings for their in- and out-group, but this difference is less pronounced ((M)_Opp_ FRA = 2.65, (M)_Opp_ GER = 2.95, (M)_Opp_ ITA = 3.18, (M)_Opp_ SPA = 2.93, (M)_Opp_ SWI = 2.87, (M)_Opp_ UK = 3.02). All differences in affective polarization are significant at the 95% level. The highest levels of affective polarization are found in Germany and Spain among supporters and in Italy and the United Kingdom among opponents. Both groups express affective polarization regarding COVID-19 vaccination, but it is stronger among the pro- than among the anti-vaccination group. As expected, affective polarization is asymmetric, implying that both in-group attachment and out-group dislike are stronger among the pro-vaccination group than among the anti-vaccination group.

### B. Measuring opinion-based affective polarization regarding COVID-19 vaccination using character trait ratings

To further evaluate our findings, we used a second common measure of affective polarization: character trait ratings^14,17,40^. Here, respondents from the pro-vaccination camp and the anti-vaccination camp (as coded above) are asked to rate various character traits of the two different groups. Although trait ratings are a typical measure of affective polarization, they reflect more than just negative affect but also shed light on the perceived stereotypical appearance of a group^9^. In this vein, these trait ratings allow us to identify whether respondents assign negative or positive characteristics to their respective in-group and out-group. Research has shown that trait ratings and thermometer scores, although conceptually somewhat distinct, correlate fairly well with each other and show little systematic differences^40^. In our full sample, the affective polarization scores for both measures correlate at (r)_Sample_ = 0.62 (r)_Sample_ FRA = 0. 61, (r)_Sample_ GER = 0.64, (r)_Sample_ ITA = 0.67, (r)_Sample_ SPA = 0.57, (r)_Sample_ SWI = 0.59, (r)_Sample_ UK = 0.65).

In our study, we asked respondents to rate the extent to which two positive character traits (openness to compromise and critical thinking) and two negative character traits (selfishness and narrow-mindedness) apply to a) supporters of COVID-19 vaccination and b) opponents of COVID-19 vaccination on a scale from 1 (“does not apply at all”) to 5 (“fully applies”). In addition to the assigned values, we also calculated the absolute differences between the scores assigned to the in- and the out-group for each trait. Subsequently, we combined these differences into an additive score for affective polarization ((M)_Sample_ FRA = 1.72, (M)_Sample_ GER = 2.08, (M)_Sample_ ITA = 1.92, (M)_Sample_ SPA = 1.99, (M)_Sample_ SWI = 1.87, (M)_Sample_ UK = 2.01).

**Figure 5:**
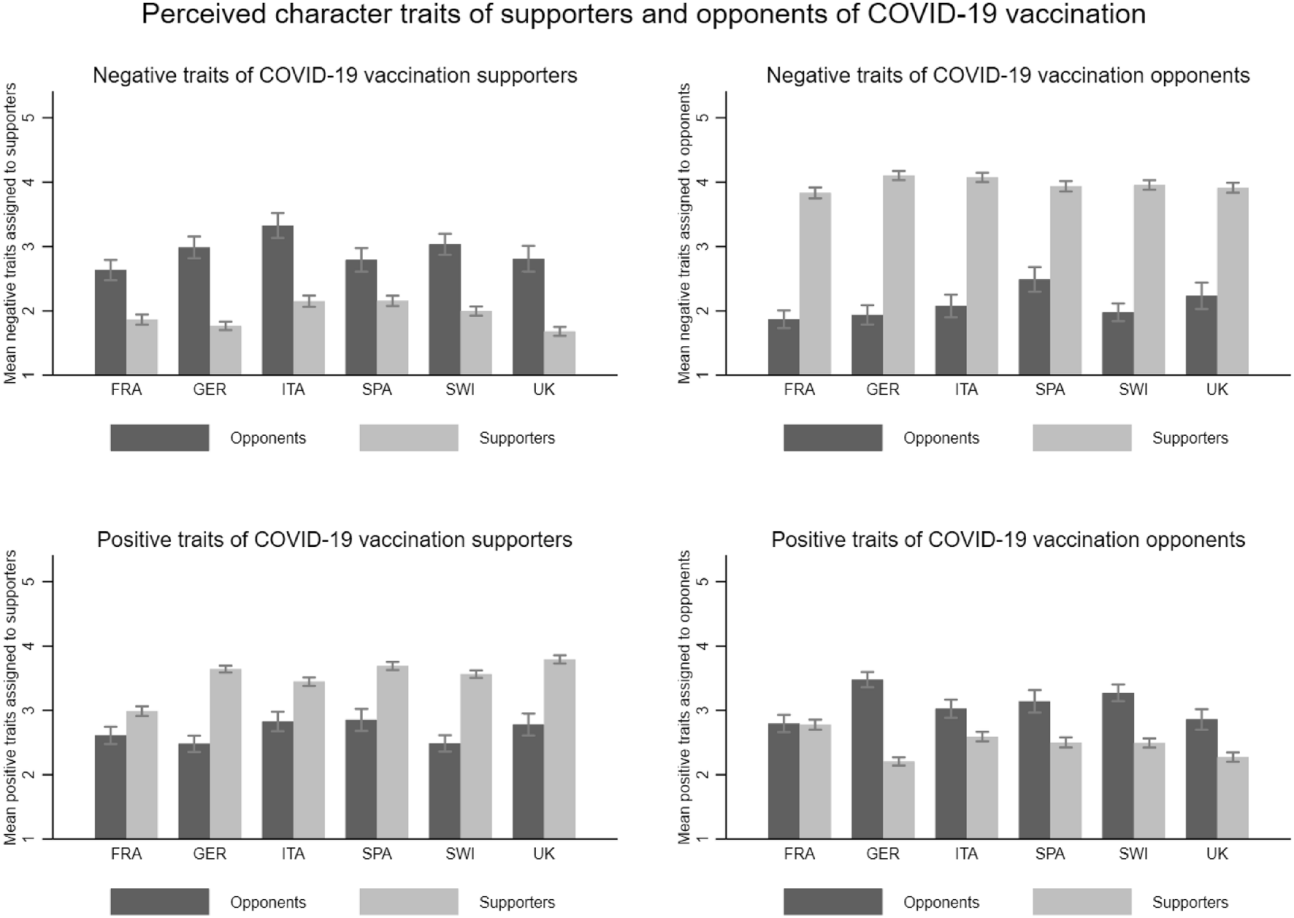
Perceived character traits of COVID-19 vaccination supporters and opponents by group and country. Notes: Figure 5 shows the mean perceived character traits for vaccination supporters and opponents separated by group and country, with 95% confidence intervals. For example, in the top left-hand panel for France: In France, vaccination opponents perceive vaccination supporters to have negative traits with an average of 2.63 while vaccination supporters perceive vaccination supporters to have negative traits with an average of 1.87 on a scale of 1–5. The difference is statistically significant.

This alternative measure reveals a similar picture of affective polarization regarding COVID-19 vaccination as the feeling thermometer. Figure 5 is analogous to Fig. 3 and shows the character trait ratings for supporters and opponents of COVID-19 vaccination by group and country. For the sake of readability, we combine the two positive and negative traits each (see supplementary material, section D, Fig. 1 for the individual character trait ratings). The upper panel of Fig. 5 shows the ratings of the two negative traits combined. As we can see in the upper left panel, supporters do not believe that other supporters are selfish and narrow-minded ((M)_Supp_ FRA = 1.87, (M)_Supp_ GER = 1.77, (M)_Supp_ ITA = 2.15, (M)_Supp_ SPA = 2.16, (M)_Supp_ SWI = 2.00, (M)_Supp_ UK = 1.68). Conversely, opponents tend to assign these negative traits to supporters ((M)_Opp_ FRA = 2.63, (M)_Opp_ GER = 3.00, (M)_Opp_ ITA = 3.33, (M) _Opp_ SPA = 2.79, (M)_Opp_ SWI = 3.04, (M)_Opp_ UK = 2.81). The opposite picture emerges when we look at the upper right panel: Consistent with in-group favoritism, opponents do not believe that other opponents are selfish or narrow-minded ((M)_Opp_ FRA = 1.87, (M)_Opp_ GER = 1.94, (M)_Opp_ ITA = 2.08, (M)_Opp_ SPA = 2.49, (M)_Opp_ SWI = 1.98, (M)_Opp_ UK = 2.23). Supporters, however, believe that opponents are selfish and narrow-minded ((M)_Supp_ FRA = 3.83, (M)_Supp_ GER = 4.10, (M)_Supp_ ITA = 4.08, (M)_Supp_ SPA = 3.94, (M)_Supp_ SWI = 3.96, (M)_Supp_ UK = 3.91). All differences are significant at the 95% level.

The lower part of Fig. 5 shows the ratings of the positive traits combined. Here, an analogous but less consistent trend is observed compared to the ratings of the negative traits. Supporters assign positive traits to their in-group ((M)_Supp_ FRA = 2.99, (M)_Supp_ GER = 3.64, (M)_Supp_ ITA = 3.45, (M)_Supp_ SPA = 3.69, (M)_Supp_ SWI = 3.56, (M)_Supp_ UK = 3.79). Conversely, opponents do not ascribe these traits to supporters ((M)_Opp_ FRA = 2.61, (M)_Opp_ GER = 2.48, (M)_Opp_ ITA = 2.83, (M)_Opp_ SPA = 2.85, (M)_Opp_ SWI = 2.49, (M)_Opp_ UK = 2.78). All differences between supporters and opponents are significant at the 95% level.

Opponents see themselves as more open to compromise and able to think critically ((M)_Opp_ FRA = 2.80, (M)_Opp_ GER = 3.48, (M)_Opp_ ITA = 3.03, (M)_Opp_ SPA = 3.14, (M)_Opp_ SWI = 3.27, (M)_Opp_ UK = 2.86). Yet, supporters do not think that these positive traits apply to opponents ((M)_Supp_ FRA = 2.78, (M)_Supp_ GER = 2.21, (M)_Supp_ ITA = 2.59, (M)_Supp_ SPA = 2.50, (M)_Supp_ SWI = 2.49, (M)_Supp_ UK = 2.27). All differences are significant at the 95% level, except those for France.

**Figure 6:**
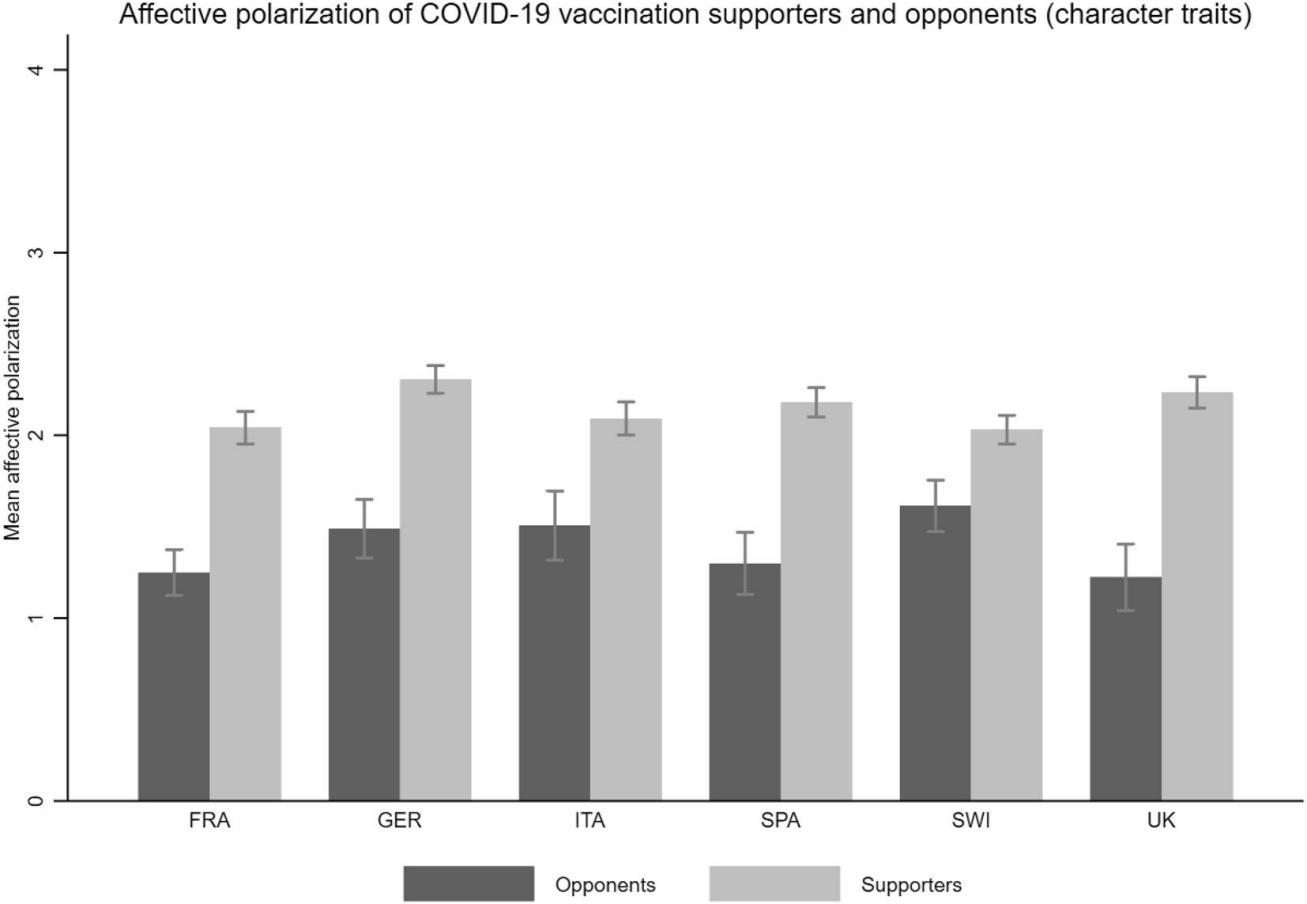
Affective polarization among COVID-19 vaccination supporters and opponents. Notes: Figure 6 shows the mean level of affective polarization (according to the character trait ratings) by group and country, with 95% confidence intervals. Reading example: In France, vaccination opponents have an average affective polarization of 1.25, while vaccination supporters have an affective polarization of 2.04 on a scale from 1 to 5. The difference is statistically significant.

Although less pronounced, Fig. 5 provides further evidence that supporters and opponents of COVID-19 vaccination tend to view their in-group positively and their out-group negatively. Figure 6, which shows the absolute difference in character trait ratings by group and country, corroborates these observations. While both groups show a difference in ascribed character traits toward the in-group and the out-group, the average difference is again slightly but statistically significantly (at the 95% level) greater among vaccination supporters ((M)_Supp_ FRA = 2.04, (M)_Supp_ GER = 2.31, (M)_Supp_ ITA = 2.09, (M)_Supp_ SPA = 2.18, (M)_Supp_ SWI = 2.03, (M)_Supp_ UK = 2.24) than among vaccination opponents ((M)_Opp_ FRA = 1.25, (M)_Opp_ GER = 1.49, (M)_Opp_ ITA = 1.51, (M)_Opp_ SPA = 1.23, (M)_Opp_ SWI = 1.61, (M)_Opp_ UK = 1.22).

Overall, our data suggest a divide around people’s opinions on COVID-19 vaccination. Supporters and opponents dislike each other and tend to attribute positive traits to their in-group and negative traits to the respective out-group. Thus, our data indicates the presence of opinion-based affective polarization regarding COVID-19 vaccination in six European democracies in early 2022. Importantly, however, this affective polarization is asymmetric, as the pro-vaccination group tends to be more polarized than the anti-vaccination group. Naturally, this finding raises the question of potential correlates of this form of polarization. As a first step in this direction, exploratory analyses reported in the supplementary material, section E, Figs. 2–4 show that older age, lower social trust, higher levels of conscientiousness, and a general support for COVID-19 vaccination are associated with higher levels of opinion-based affective polarization regarding COVID-19 vaccination.

## Discussion

In this article, we shed light on the social fabric during the COVID-19 pandemic. Specifically, we extend research on affective polarization by focusing on opinion-based rather than partisan affective polarization. While partisan affective polarization has been described as a major challenge to democracy, partisan identities are not the only political identities that matter for intergroup hostility. Using COVID-19 vaccination as a prime example, we show that this issue formed the basis of an affective divide in the population in six European democracies in 2022. Applying both thermometer scores and trait ratings reflecting the perceived stereotypical appearance of a group, we observe an asymmetric affective polarization regarding COVID-19 vaccination in all six countries. While both supporters and opponents of COVID-19 vaccination show affective polarization, this polarization is higher among the pro-vaccination group^1,2^.

This report sets the stage for further research on opinion-based affective polarization that goes beyond the limitations of this study, which we address in the following. First, we operate with cross-sectional survey data and correlational analyses that are limited to a specific point in time. Experimental and longitudinal studies of group formation around COVID-19 vaccination would be a promising next step, particularly to see whether this division persists after the issue has faded in salience. Second, COVID-19 vaccination is a specific issue, raising the question of what other issues might serve as the basis for opinion-based group identities and subsequent affective polarization^15,16,33^. Third, although the finding of asymmetric affective polarization is consistent across the countries studied, some individual character traits show less consistency than others in the individual countries. This applies, for example, to the character trait of critical thinking in Italy and France. Furthermore, our analyses are limited to the character traits that have been examined in previous research on affective polarization. Future research is advised to expand the inventory and explore other characteristics such as honesty or trustworthiness^9^. In general, we must acknowledge that the expression of stereotypes seems to be less prevalent than direct dislike between the groups, as evidenced by the thermometer scores.

In addition, future studies could evaluate additional factors that may contribute to affective polarization, such as a tendency toward analytical thinking, different worldviews, and ideological orientations (e.g., hierarchy–egalitarianism and individualism–communitarianism). In addition, statistical techniques such as latent profile or class analysis might provide more insight into which groups in society express higher levels of dislike and stereotypical thinking about their out-groups. Fourth, future research could examine the consequences of opinion-based affective polarization and compare them to the presumed consequences of partisan affective polarization, particularly in terms of democratic norms, social cohesion, and health behavior^18,19,22,23^.

Despite these limitations, our findings have important policy implications regarding the challenges posed by infectious diseases. First, high levels of opinion-based affective polarization undermine efforts to implement public health measures and limit the role civil society can play in overcoming a pandemic. In-group favorability and out-group hostility put a strain on democratic societies, which in times of pandemic threat may undermine a unified and coherent public response to contain the spread of infectious diseases. Second, affective polarization may cause individuals to selectively engage with information that aligns with their pre-existing beliefs, further reinforcing misperceptions^41–45^. Such misperceptions often contradict evidence-based public information and hamper public responses to infectious diseases. Third, social cohesion suffers from affective polarization and the division into an “us-versus-them” mentality. Yet, public health crises require a collective response, which is threatened by affective divisions that polarize civil society. Addressing affective polarization is a difficult task that will require a refinement of traditional public health strategies. Intergroup contact, social outreach, and the creation and communication of common overarching identities may prove useful in reducing affective polarization and fostering a more unified response to public health threats in the future^46–49^.

## Methods

### Methods

The data were collected between January 25 and March 8, 2022, in France, Germany, Italy, Spain, Switzerland, and the United Kingdom. The survey was conducted by the German survey company SurveyEngine, using quota-sampling regarding gender, age, and education (and language in the case of Switzerland) yielding around 1000 respondents per country. During the data collection, the pandemic was still very present due to the emergence of the Omicron variant. More importantly, although vaccines were widely available, there were heated debates about the value and consequences of COVID-19 vaccination.

### Participants

Our survey included n = 6379 participants (France: n = 1078, Germany: n = 1080, Italy: n = 1053, Spain: n = 1013, Switzerland: n = 1114, UK: n = 1041). Participants were 18 to 91 years old (M = 48.04 years, S.D. = 15.87 and 49% were female. Education levels are distributed as follows: 25% had primary or lower secondary education (FRA: 20%, GER: 15%, ITA: 37%, SPA: 38%, SWI: 11%, UK: 33%), 40% had upper, post-secondary education (FRA: 44%, GER: 56%, ITA: 43%, SPA: 24%, SWI: 47%, UK: 21%), and 35% had a tertiary education (FRA: 36%, GER: 29%, ITA: 20%, SPA: 37%, SWI: 41%, UK: 46%).

### Ethical approval

Ethical clearance for the study was obtained from the Ethics Committee of the Faculty of Business Administration, Economics and Social Sciences of the University of Bern with the project number 092020. Participants provided informed consent and received a small compensation from the survey company for their participation.

### Reporting summary

Further information on the research design is available in the Nature Research Reporting Summary linked to this article.

### Materials and measures

#### Position on COVID-19 vaccination

Respondents were asked what they think about COVID-19 vaccinations to contain the COVID-19 pandemic. Answers were recorded on an 11-point scale, ranging from 0 “I completely reject them” to 10 “I completely support them”. In the analyses, we excluded respondents who indicated a value of 5 as they expressed a neutral position on the issue, making it unlikely that they had an opinion-based identity. Thus, we dichotomized this variable and classified respondents who indicated a value from 0 to 4 as opponents of COVID-19 vaccination and those who indicated a value from 6 to 10 as supporters of COVID-19 vaccination.

#### Thermometer ratings

Respondents were asked to rate their feelings toward two groups: (a) people who get vaccinated (b) people who do not get vaccinated. Respondents were asked to describe their feelings using a thermometer scale ranging from − 5 to + 5, with − 5 meaning they feel very cold and negative toward the group and + 5 means they feel very sympathetic and positive toward the group. We converted this measure into a scale ranging from 0 to 10.

#### Rating of the character traits of vaccination opponents

Respondents were asked to think about people who do not want vaccinations for themselves (and others) and to rate to what extent they think the following four character traits apply to this group of people: (a) willingness to compromise, (b) narrow-mindedness, (c) selfishness, and (d) critical thinking. Answers were recorded on a 5-point scale ranging from 1 “does not apply at all” to 5 “fully applies”.

#### Rating of the character traits of vaccination supporters

Respondents were asked to think about people who want vaccinations for themselves (and others) and to rate to what extent they think the following four character traits apply to this group of people: (a) willingness to compromise, (b) narrow-mindedness, (c) selfishness, and (d) critical thinking. Answers were recorded on a 5-point scale, ranging from 1 “does not apply at all” to 5 “fully applies”.

#### Social trust

Social trust was measured through the generalized trust question, which asks respondents whether they think most people can be trusted or whether one can’t be too careful in dealing with people. Answers were recorded on a 7-point scale, ranging from 1 “you can’t be too careful in dealing with people” to 7 “most people can be trusted”.

#### Political trust

Political trust was measured asking respondents whether they trust the following political institutions: (a) the national government, (b) the national parliament, and (c) national politicians. Answers were recorded on a 5-point scale, ranging from 1 “strongly disagree” to 5 “strongly agree”. The corresponding items were combined into an aggregate measure using the arithmetic mean.

#### Left–right self-placement

Left–right self-placement was assessed using the following question: “In political matters, people talk of ‘left’ and ‘right’. How would you place your views on this scale, generally speaking?” Answers were recorded on an 11-point scale ranging from 0 “left” to 10 “right”.

#### Big Five personality traits

To measure the Big Five personality traits, we used the widespread Ten-Item Personality Inventory (TIPI) including the adjectives (a) extraverted, enthusiastic (extraversion); (b) critical, quarrelsome (agreeableness, reverse-coded); (c) dependable, self-disciplined (conscientiousness); (d) anxious, easily upset (neuroticism); (e) open to new experiences, complex (openness to experience); (f) reserved, quiet (extraversion, reverse-coded); (g) sympathetic, warm (agreeableness); (h) disorganized, careless (conscientiousness, reverse-coded); (i) calm, emotionally stable (neuroticism, reverse-coded); and (j) conventional, uncreative (openness to experience, reverse-coded). Answers were recorded on a 5-point scale ranging from 1 “strongly disagree” to 5 “strongly agree”. The corresponding items were combined into the five personality traits using arithmetic means.

### Supplementary Information

Supplementary Information.

## Data Availability

Materials and data will be made available via the OSF at https://osf.io/ax9m4/?view_only=05b3a6c20fe644b285f6d80453728c5a.

## Code availability

The data analysis script (including reported and supplementary analyses) will be made available via the OSF at https://osf.io/ax9m4/?view_only=05b3a6c20fe644b285f6d80453728c5a.
