# Supplementary material for: Asymmetric affective polarization regarding COVID-19 vaccination in six European countries
Source: Sci Rep. 2024 Jul 10;14:15919. doi: 10.1038/s41598-024-66756-w (PMC11237099; doi:10.1038/s41598-024-66756-w)
Supplement: Supplementary file 1 — Supplementary Information. [file 41598_2024_66756_MOESM1_ESM.docx]

**Online supplementary material to: Asymmetric affective polarization regarding COVID-19 vaccination in six European countries**

**Supplement A Description of the survey and descriptive statistics**

Table 1 Detailed description of the survey

| Period | January 25, 2022 – March 08, 2022 |
| --- | --- |
| Target population | Residents aged 18 years or older in Germany, France, Italy, Switzerland, Spain, and the United Kingdom |
| Survey mode | Online |
| Sample size | 6,379 respondents (target sample size: 1,000 per country) |
| Quotas | Age, Sex, Education (language for Switzerland) |
| Sampling | SurveyEngine access panel |
| Interview language | German, French, Italian, Spanish, English |
| Response rate | 19.16% (RR5/6) |
| Institute | SurveyEngine |

Table 2 Distribution of sex

| Country | | Sex |  |
| --- | --- | --- | --- |
|  |  | Female | Male |
| France | | 50.4% | 49.6% |
| Germany | | 49.4% | 50.6% |
| Italy | | 49.2% | 50.8% |
| Spain | | 49.9% | 50.2% |
| Switzerland | | *48.5%* | *51.5%* |
| United Kingdom | | *49.0%* | *51.0%* |

*Notes: Deviation from quotas; italics = more than 1%, bold = more than 2%)*

Table 3 Distribution of age

| Country | | Age |  |  |  |
| --- | --- | --- | --- | --- | --- |
|  |  | 18-30 | 30-45 | 45-60 | 60+ |
| France | | 19.4% | **21.2%** | 26.8% | *32.6%* |
| Germany | | 18.4% | *22.8%* | 25.4% | **33.4%** |
| Italy | | 19.9% | 23.5% | 26.7% | 30.0% |
| Spain | | **21.7%** | 23.2% | 26.3% | **28.8%** |
| Switzerland | | 18.9% | *25.6%* | **28.6%** | **27.1%** |
| United Kingdom | | 25.3% | *22.4%* | *25.0%* | 27.4% |

*Notes: Deviation from quotas; yellow more than 1%, red more than 2%)*

Table 4 Distribution of education

| Country | | Education |  |  |
| --- | --- | --- | --- | --- |
|  |  | Below Upper Secondary | Upper Secondary  and Post-secondary non-tertiary | Tertiary |
| France | | 20.0% | **44.2%** | *35.8%* |
| Germany | | *15.4%* | *55.7%* | 29.0% |
| Italy | | *37.0%* | 42.5% | *20.5%* |
| Spain | | 38.8% | 24.0% | 37.2% |
| Switzerland | | 11.2% | **47.7%** | **41.1%** |
| United Kingdom | | 33.0% | 21.1% | 45.8% |

*Notes: Deviation from quotas; yellow more than 1%, red more than 2%*

Table 5 Descriptive statistics estimation sample in France

| **France** | | | | | |
| --- | --- | --- | --- | --- | --- |
|  | N | Mean | SD | Min | Max |
| Affective Polarization (Character trait) | 821 | 1.69 | 1.22 | 0 | 4 |
| Affective Polarization (Thermometer) | 821 | 5.06 | 4.00 | 0 | 10 |
| Age | 821 | 48.47 | 15.57 | 19 | 85 |
| Sex | 821 | .51 | .50 | 0 | 1 |
| *Female (0)* | 405 |  |  |  |  |
| *Male (1)* | 416 |  |  |  |  |
| Education | 821 | 2.17 | .72 | 1 | 3 |
| *Primary & lower secondary (1)* | 153 |  |  |  |  |
| *Upper, post-secondary (2)* | 373 |  |  |  |  |
| *Tertiary (3)* | 295 |  |  |  |  |
| Children | 821 | .36 | .48 | 0 | 1 |
| *No (0)* | 526 |  |  |  |  |
| *Yes (1)* | 295 |  |  |  |  |
| Income situation | 821 | 2.84 | .99 | 1 | 5 |
| Self-rated health | 821 | 3.71 | .87 | 1 | 5 |
| Type of community | 821 | 2.55 | 1.41 | 1 | 5 |
| *Rural area (1)* | 260 |  |  |  |  |
| *Small town (2)* | 184 |  |  |  |  |
| *Middle-sized town (3)* | 165 |  |  |  |  |
| *Suburb (4)* | 90 |  |  |  |  |
| *Big City (5)* | 122 |  |  |  |  |
| Left-right self-placement | 821 | 5.37 | 2.42 | 0 | 10 |
| Political trust | 821 | 3.11 | 1.45 | 1 | 7 |
| Social trust | 821 | 3.11 | 1.61 | 1 | 7 |
| Position on vaccination | 821 | 6.94 | 3.25 | 0 | 10 |
| Big Five: Extraversion | 821 | 2.75 | .77 | 1 | 5 |
| Big Five: Agreeableness | 821 | 3.69 | .76 | 1 | 5 |
| Big Five: Conscientiousness | 821 | 3.95 | .81 | 1 | 5 |
| Big Five: Neuroticism | 821 | 2.71 | .84 | 1 | 5 |
| Big Five: Openness | 821 | 3.29 | .73 | 1 | 5 |
| Observations | 821 |  |  |  |  |

Notes: The number of observations is based on the regression analyses of figure 7. The lower number of observations is due to the exclusion of respondents who did not answer questions about whether they have children or not as well as a few missing responses for income situation and self-rated health. Models without the variable on children provide substantially the same results.

Table 6 Descriptive statistics estimation sample in Germany

| **Germany** | | | | | |
| --- | --- | --- | --- | --- | --- |
|  | N | Mean | SD | Min | Max |
| Affective Polarization (Character trait) | 755 | 2.09 | 1.18 | 0 | 4 |
| Affective Polarization (Thermometer) | 755 | 6.11 | 3.77 | 0 | 10 |
| Age | 755 | 48.82 | 16.19 | 18 | 82 |
| Sex | 755 | .49 | .50 | 0 | 1 |
| *Female (0)* | 384 |  |  |  |  |
| *Male (1)* | 371 |  |  |  |  |
| Education | 755 | 2.16 | .67 | 1 | 3 |
| *Primary & lower secondary (1)* | 120 |  |  |  |  |
| *Upper, post-secondary (2)* | 393 |  |  |  |  |
| *Tertiary (3)* | 242 |  |  |  |  |
| Children | 755 | .25 | .43 | 0 | 1 |
| *No (0)* | 567 |  |  |  |  |
| *Yes (1)* | 188 |  |  |  |  |
| Income situation | 755 | 3.33 | .99 | 1 | 5 |
| Self-rated health | 755 | 3.69 | .93 | 1 | 5 |
| Type of community | 755 | 2.90 | 1.49 | 1 | 5 |
| *Rural area (1)* | 172 |  |  |  |  |
| *Small town (2)* | 186 |  |  |  |  |
| *Middle-sized town (3)* | 119 |  |  |  |  |
| *Suburb (4)* | 102 |  |  |  |  |
| *Big City (5)* | 176 |  |  |  |  |
| Left-right self-placement | 755 | 4.74 | 1.79 | 0 | 10 |
| Political trust | 755 | 3.62 | 1.62 | 1 | 7 |
| Social trust | 755 | 3.71 | 1.63 | 1 | 7 |
| Position on vaccination | 755 | 7.87 | 3.25 | 0 | 10 |
| Big Five: Extraversion | 755 | 2.93 | .90 | 1 | 5 |
| Big Five: Agreeableness | 755 | 3.72 | .74 | 1 | 5 |
| Big Five: Conscientiousness | 755 | 4.123 | .80 | 1 | 5 |
| Big Five: Neuroticism | 755 | 2.34 | .90 | 1 | 5 |
| Big Five: Openness | 755 | 3.62 | .78 | 1.5 | 5 |
| Observations | 755 |  |  |  |  |

Notes: The number of observations is based on the regression analyses of figure 7. The lower number of observations is due to the exclusion of respondents who did not answer questions about whether they have children or not as well as a few missing responses for income situation and self-rated health. Models without the variable on children provide substantially the same results.

Table 7 Descriptive statistics estimation sample in Italy

| **Italy** | | | | | |
| --- | --- | --- | --- | --- | --- |
|  | N | Mean | SD | Min | Max |
| Affective Polarization (Character trait) | 892 | 1.89 | 1.35 | 0 | 4 |
| Affective Polarization (Thermometer) | 892 | 6.08 | 4.10 | 0 | 10 |
| Age | 892 | 47.37 | 16.05 | 18 | 91 |
| Sex | 892 | .51 | .50 | 0 | 1 |
| *Female (0)* | 436 |  |  |  |  |
| *Male (1)* | 456 |  |  |  |  |
| Education | 892 | 1.83 | .74 | 1 | 3 |
| *Primary & lower secondary (1)* | 332 |  |  |  |  |
| *Upper, post-secondary (2)* | 380 |  |  |  |  |
| *Tertiary (3)* | 180 |  |  |  |  |
| Children | 892 | .31 | .46 | 0 | 1 |
| *No (0)* | 615 |  |  |  |  |
| *Yes (1)* | 277 |  |  |  |  |
| Income situation | 892 | 2.74 | 1.02 | 1 | 5 |
| Self-rated health | 892 | 3.70 | .974 | 1 | 5 |
| Type of community | 892 | 2.66 | 1.39 | 1 | 5 |
| *Rural area (1)* | 227 |  |  |  |  |
| *Small town (2)* | 224 |  |  |  |  |
| *Middle-sized town (3)* | 218 |  |  |  |  |
| *Suburb (4)* | 69 |  |  |  |  |
| *Big City (5)* | 154 |  |  |  |  |
| Left-right self-placement | 892 | 5.37 | 2.68 | 0 | 10 |
| Political trust | 892 | 3.28 | 1.64 | 1 | 7 |
| Social trust | 892 | 3.70 | 1.72 | 1 | 7 |
| Position on vaccination | 892 | 7.78 | 2.94 | 0 | 10 |
| Big Five: Extraversion | 892 | 2.83 | .82 | 1 | 5 |
| Big Five: Agreeableness | 892 | 3.72 | .83 | 1 | 5 |
| Big Five: Conscientiousness | 892 | 3.78 | .87 | 1 | 5 |
| Big Five: Neuroticism | 892 | 2.62 | .85 | 1 | 5 |
| Big Five: Openness | 892 | 3.110 | .66 | 1 | 5 |
| Observations | 892 |  |  |  |  |

Notes: The number of observations is based on the regression analyses of figure 7. The lower number of observations is due to the exclusion of respondents who did not answer questions about whether they have children or not as well as a few missing responses for income situation and self-rated health. Models without the variable on children provide substantially the same results.

Table 8 Descriptive statistics estimation sample in Spain

| **Spain** | | | | | |
| --- | --- | --- | --- | --- | --- |
|  | N | Mean | SD | Min | Max |
| Affective Polarization (Character trait) | 888 | 1.99 | 1.21 | 0 | 4 |
| Affective Polarization (Thermometer) | 888 | 6.42 | 3.8 | 0 | 10 |
| Age | 888 | 47.11 | 14.36 | 18 | 79 |
| Sex | 888 | .50 | .50 | 0 | 1 |
| *Female (0)* | 442 |  |  |  |  |
| *Male (1)* | 446 |  |  |  |  |
| Education | 888 | 1.99 | .87 | 1 | 3 |
| *Primary & lower secondary (1)* | 340 |  |  |  |  |
| *Upper, post-secondary (2)* | 219 |  |  |  |  |
| *Tertiary (3)* | 329 |  |  |  |  |
| Children | 888 | .29 | .45 | 0 | 1 |
| *No (0)* | 631 |  |  |  |  |
| *Yes (1)* | 257 |  |  |  |  |
| Income situation | 888 | 2.90 | 1.04 | 1 | 5 |
| Self-rated health | 888 | 3.50 | .85 | 1 | 5 |
| Type of community | 888 | 3.08 | 1.54 | 1 | 5 |
| *Rural area (1)* | 198 |  |  |  |  |
| *Small town (2)* | 142 |  |  |  |  |
| *Middle-sized town (3)* | 217 |  |  |  |  |
| *Suburb (4)* | 53 |  |  |  |  |
| *Big City (5)* | 278 |  |  |  |  |
| Left-right self-placement | 888 | 4.23 | 2.58 | 0 | 10 |
| Political trust | 888 | 2.70 | 1.37 | 1 | 7 |
| Social trust | 888 | 3.76 | 1.65 | 1 | 7 |
| Position on vaccination | 888 | 7.98 | 2.80 | 0 | 10 |
| Big Five: Extraversion | 888 | 2.89 | .75 | 1 | 5 |
| Big Five: Agreeableness | 888 | 3.27 | .56 | 1.5 | 5 |
| Big Five: Conscientiousness | 888 | 3.79 | .75 | 1.5 | 5 |
| Big Five: Neuroticism | 888 | 2.57 | .82 | 1 | 5 |
| Big Five: Openness | 888 | 3.41 | .80 | 1 | 5 |
| Observations | 888 |  |  |  |  |

Notes: The number of observations is based on the regression analyses of figure 7. The lower number of observations is due to the exclusion of respondents who did not answer questions about whether they have children or not as well as a few missing responses for income situation and self-rated health. Models without the variable on children provide substantially the same results.

Table 9 Descriptive statistics estimation sample in Switzerland

| **Switzerland** | | | | | |
| --- | --- | --- | --- | --- | --- |
|  | N | Mean | SD | Min | Max |
| Affective Polarization (Character trait) | 785 | 1.88 | 1.15 | 0 | 4 |
| Affective Polarization (Thermometer) | 785 | 5.28 | 3.82 | 0 | 10 |
| Age | 785 | 46.90 | 16.05 | 18 | 86 |
| Sex | 785 | .54 | .50 | 0 | 1 |
| *Female (0)* | 362 |  |  |  |  |
| *Male (1)* | 423 |  |  |  |  |
| Education | 785 | 2.31 | .67 | 1 | 3 |
| *Primary & lower secondary (1)* | 90 |  |  |  |  |
| *Upper, post-secondary (2)* | 359 |  |  |  |  |
| *Tertiary (3)* | 336 |  |  |  |  |
| Children | 785 | .31 | .46 | 0 | 1 |
| *No (0)* | 539 |  |  |  |  |
| *Yes (1)* | 246 |  |  |  |  |
| Income situation | 785 | 3.26 | 1.04 | 1 | 5 |
| Self-rated health | 785 | 3.97 | .91 | 1 | 5 |
| Type of community | 785 | 2.38 | 1.41 | 1 | 5 |
| *Rural area (1)* | 310 |  |  |  |  |
| *Small town (2)* | 158 |  |  |  |  |
| *Middle-sized town (3)* | 122 |  |  |  |  |
| *Suburb (4)* | 102 |  |  |  |  |
| *Big City (5)* | 93 |  |  |  |  |
| Left-right self-placement | 785 | 5.20 | 2.24 | 0 | 10 |
| Political trust | 785 | 4.37 | 1.35 | 1 | 7 |
| Social trust | 785 | 4.02 | 1.58 | 1 | 7 |
| Position on vaccination | 785 | 7.43 | 3.28 | 0 | 10 |
| Big Five: Extraversion | 785 | 2.91 | .80 | 1 | 5 |
| Big Five: Agreeableness | 785 | 3.75 | .70 | 1.5 | 5 |
| Big Five: Conscientiousness | 785 | 4.07 | .75 | 1 | 5 |
| Big Five: Neuroticism | 785 | 2.36 | .81 | 1 | 5 |
| Big Five: Openness | 785 | 3.55 | .75 | 1 | 5 |
| Observations | 785 |  |  |  |  |

Notes: The number of observations is based on the regression analyses of figure 7. The lower number of observations is due to the exclusion of respondents who did not answer questions about whether they have children or not as well as a few missing responses for income situation and self-rated health. Models without the variable on children provide substantially the same results.

Table 10 Descriptive statistics estimation sample in the United Kingdom

| **United Kingdom** | | | | | |
| --- | --- | --- | --- | --- | --- |
|  | N | Mean | SD | Min | Max |
| Affective Polarization (Character trait) | 807 | 2.01 | 1.32 | 0 | 4 |
| Affective Polarization (Thermometer) | 807 | 6.04 | 3.80 | 0 | 10 |
| Age | 807 | 45.84 | 16.37 | 18 | 87 |
| Sex | 807 | .50 | .50 | 0 | 1 |
| *Female (0)* | 401 |  |  |  |  |
| *Male (1)* | 406 |  |  |  |  |
| Education | 807 | 2.14 | .87 | 1 | 3 |
| *Primary & lower secondary (1)* | 254 |  |  |  |  |
| *Upper, post-secondary (2)* | 185 |  |  |  |  |
| *Tertiary (3)* | 368 |  |  |  |  |
| Children | 807 | .33 | .47 | 0 | 1 |
| *No (0)* | 541 |  |  |  |  |
| *Yes (1)* | 266 |  |  |  |  |
| Income situation | 807 | 3.14 | 1.08 | 1 | 5 |
| Self-rated health | 807 | 3.70 | .96 | 1 | 5 |
| Type of community | 807 | 2.88 | 1.34 | 1 | 5 |
| *Rural area (1)* | 166 |  |  |  |  |
| *Small town (2)* | 173 |  |  |  |  |
| *Middle-sized town (3)* | 174 |  |  |  |  |
| *Suburb (4)* | 183 |  |  |  |  |
| *Big City (5)* | 111 |  |  |  |  |
| Left-right self-placement | 807 | 4.90 | 2.07 | 0 | 10 |
| Political trust | 807 | 3.16 | 1.50 | 1 | 7 |
| Social trust | 807 | 3.95 | 1.64 | 1 | 7 |
| Position on vaccination | 807 | 8.17 | 2.86 | 0 | 10 |
| Big Five: Extraversion | 807 | 2.74 | .92 | 1 | 5 |
| Big Five: Agreeableness | 807 | 3.66 | .81 | 1 | 5 |
| Big Five: Conscientiousness | 807 | 3.88 | .84 | 1 | 5 |
| Big Five: Neuroticism | 807 | 2.63 | .95 | 1 | 5 |
| Big Five: Openness | 807 | 3.41 | .78 | 1 | 5 |
| Observations | 807 |  |  |  |  |

Notes: The number of observations is based on the regression analyses of figure 7. The lower number of observations is due to the exclusion of respondents who did not answer questions about whether they have children or not as well as a few missing responses for income situation and self-rated health. Models without the variable on children provide substantially the same results.

**Supplement B Additional analyses of vaccination groups**

Table 11 Linear regression model on trust in government

|  | Full Sample | France | Germany | Italy | Spain | Switzerland | UK |
| --- | --- | --- | --- | --- | --- | --- | --- |
| *Position on COVID-19 vaccination* |  |  |  |  |  |  |  |
| Supporter | 1.25*** (0.05) | 1.27*** (0.11) | 1.62*** (0.13) | 1.60*** (0.12) | 0.62*** (0.16) | 1.55*** (0.12) | 0.33* (0.16) |
| *Sex* |  |  |  |  |  |  |  |
| Male | 0.15*** (0.04) | 0.02 (0.10) | 0.09 (0.10) | 0.49*** (0.11) | 0.09 (0.11) | 0.12 (0.09) | -0.02 (0.10) |
| *Education* |  |  |  |  |  |  |  |
| Upper, post-secondary | -0.22*** (0.06) | -0.21 (0.15) | 0.00 (0.15) | -0.33** (0.13) | -0.15 (0.14) | -0.13 (0.16) | 0.09 (0.14) |
| Tertiary | -0.00 (0.06) | 0.15 (0.15) | 0.41* (0.16) | -0.06 (0.15) | -0.08 (0.13) | -0.06 (0.17) | 0.11 (0.12) |
| Income situation | 0.19*** (0.02) | 0.15** (0.06) | 0.27*** (0.05) | 0.39*** (0.06) | 0.02 (0.05) | 0.12* (0.05) | 0.14** (0.05) |
| Self-rated health | 0.08** (0.03) | 0.13* (0.06) | 0.14* (0.06) | -0.23*** (0.06) | 0.19** (0.07) | 0.13* (0.06) | 0.11* (0.06) |
| Left-right self-placement | 0.00 (0.01) | 0.02 (0.02) | -0.09** (0.03) | -0.02 (0.02) | -0.18*** (0.02) | 0.02 (0.02) | 0.32*** (0.03) |
|  |  |  |  |  |  |  |  |
| Constant | 1.24*** (0.13) | 1.04*** (0.28) | 1.21*** (0.30) | 1.72*** (0.30) | 2.25*** (0.31) | 2.36*** (0.30) | 0.23 (0.27) |
| Observations | 5774 | 934 | 988 | 958 | 924 | 1006 | 964 |
| Country fixed-effects | ✓ | - | - | - | - | - | - |
| *R*^2^ | 0.214 | 0.141 | 0.221 | 0.217 | 0.103 | 0.199 | 0.177 |

Robust standard errors in parentheses

+ p<0.10, * p<0.05, ** p<0.01, *** p<0.001

**Supplement C T-test of differences between supporters and opponents**

Table 12 T-tests of thermometer ratings and affective polarization between supporters and opponents by country

|  | France | Germany | Italy | Spain | Switzerland | UK |
| --- | --- | --- | --- | --- | --- | --- |
|  |  |  |  |  |  |  |
| Thermometer rating towards vaccination supporters | -4.037^***^ | -4.753^***^ | -4.796^***^ | -4.140^***^ | -4.288^***^ | -4.577^***^ |
|  | (-24.31) | (-32.02) | (-25.98) | (-19.78) | (-28.32) | (-24.90) |
|  |  |  |  |  |  |  |
| Thermometer rating towards vaccination opponents | 4.097^***^ | 4.664^***^ | 4.496^***^ | 4.017^***^ | 4.120^***^ | 3.645^***^ |
|  | (20.15) | (23.19) | (16.80) | (16.53) | (20.39) | (14.86) |
|  |  |  |  |  |  |  |
| Affective Polarization (Thermometer) | -3.977^***^ | -4.220^***^ | -3.825^***^ | -4.413^***^ | -3.490^***^ | -3.837^***^ |
|  | (-15.30) | (-15.63) | (-11.67) | (-13.82) | (-13.50) | (-12.27) |
| *N* | 949 | 1003 | 988 | 949 | 1042 | 986 |

*t* statistics in parentheses

^*^ *p* < 0.05, ^**^ *p* < 0.01, ^***^ *p* < 0.001

Table 13 T-tests of character traits and affective polarization between supporters and opponents by country

|  | France | Germany | Italy | Spain | Switzerland | UK |
| --- | --- | --- | --- | --- | --- | --- |
|  |  |  |  |  |  |  |
| Negative traits of supporters | 0.769^***^ | 1.219^***^ | 1.177^***^ | 0.636^***^ | 1.038^***^ | 1.128^***^ |
|  | (9.18) | (14.81) | (10.56) | (5.92) | (12.56) | (11.78) |
|  |  |  |  |  |  |  |
| Negative traits of opponents | -1.964^***^ | -2.166^***^ | -1.998^***^ | -1.448^***^ | -1.980^***^ | -1.679^***^ |
|  | (-23.13) | (-25.04) | (-21.20) | (-13.18) | (-24.81) | (-15.74) |
|  |  |  |  |  |  |  |
| Positive traits of supporters | -0.376^***^ | -1.162^***^ | -0.618^***^ | -0.839^***^ | -1.077^***^ | -1.011^***^ |
|  | (-4.91) | (-17.81) | (-7.35) | (-9.52) | (-16.28) | (-11.47) |
|  |  |  |  |  |  |  |
| Positive traits of opponents | 0.0168 | 1.271^***^ | 0.434^***^ | 0.638^***^ | 0.779^***^ | 0.587^***^ |
|  | (0.21) | (16.48) | (4.66) | (6.04) | (10.18) | (6.09) |
|  |  |  |  |  |  |  |
| Affective Polarization (Character trait) | -0.793^***^ | -0.817^***^ | -0.586^***^ | -0.882^***^ | -0.417^***^ | -1.012^***^ |
|  | (-9.13) | (-8.90) | (-5.09) | (-8.21) | (-4.91) | (-8.87) |
| *N* | 949 | 1003 | 988 | 949 | 1042 | 986 |

*t* statistics in parentheses

^*^ *p* < 0.05, ^**^ *p* < 0.01, ^***^ *p* < 0.001

**Supplement D Robustness individual character traits**

Table 14 T-tests of character traits and affective polarization between supporters and opponents by country

|  | France | Germany | Italy | Spain | Switzerland | UK |  |
| --- | --- | --- | --- | --- | --- | --- | --- |
|  |  |  |  |  |  |  |  |
| Supporters: Selfishness | 0.735^***^ | 1.154^***^ | 1.131^***^ | 0.674^***^ | 1.000^***^ | 1.015^***^ |  |
|  | (8.18) | (12.68) | (9.55) | (5.62) | (10.67) | (9.93) |  |
|  |  |  |  |  |  |  |  |
| Opponents: Selfishness | -2.122^***^ | -2.248^***^ | -2.171^***^ | -1.752^***^ | -2.161^***^ | -1.745^***^ |  |
|  | (-23.24) | (-23.92) | (-21.22) | (-14.10) | (-24.97) | (-15.30) |  |
|  |  |  |  |  |  |  |  |
| Supporters: Narrowmindedness | 0.804^***^ | 1.283^***^ | 1.223^***^ | 0.599^***^ | 1.076^***^ | 1.241^***^ |  |
|  | (8.74) | (13.98) | (10.05) | (4.85) | (11.72) | (12.21) |  |
|  |  |  |  |  |  |  |  |
| Opponents: Narrowmindedness | -1.806^***^ | -2.084^***^ | -1.825^***^ | -1.145^***^ | -1.798^***^ | -1.614^***^ |  |
|  | (-19.56) | (-22.82) | (-17.17) | (-9.25) | (-20.46) | (-14.50) |  |
|  |  |  |  |  |  |  |  |
| Supporters: Openness to compromise | -0.742^***^ | -1.415^***^ | -0.956^***^ | -1.171^***^ | -1.481^***^ | -1.274^***^ |  |
|  | (-7.44) | (-17.01) | (-8.89) | (-11.59) | (-17.86) | (-12.35) |  |
|  |  |  |  |  |  |  |  |
| Opponents: Openness to compromise | 0.374^***^ | 1.269^***^ | 0.744^***^ | 0.674^***^ | 0.850^***^ | 0.530^***^ |  |
|  | (3.79) | (14.69) | (6.55) | (5.18) | (9.21) | (4.70) |  |
|  |  |  |  |  |  |  |  |
| Supporters: Critical thinking | -0.0106 | -0.909^***^ | -0.281^*^ | -0.506^***^ | -0.673^***^ | -0.748^***^ |  |
|  | (-0.11) | (-9.55) | (-2.38) | (-3.96) | (-7.36) | (-6.39) |  |
|  |  |  |  |  |  |  |  |
| Opponents: Critical thinking | -0.340^***^ | 1.273^***^ | 0.124 | 0.602^***^ | 0.709^***^ | 0.644^***^ |  |
|  | (-3.32) | (11.89) | (1.01) | (4.46) | (7.31) | (5.54) |  |
| *N* | 949 | 1003 | 988 | 949 | 1042 | 986 |  |

*t* statistics in parentheses

^*^ *p* < 0.05, ^**^ *p* < 0.01, ^***^ *p* < 0.001


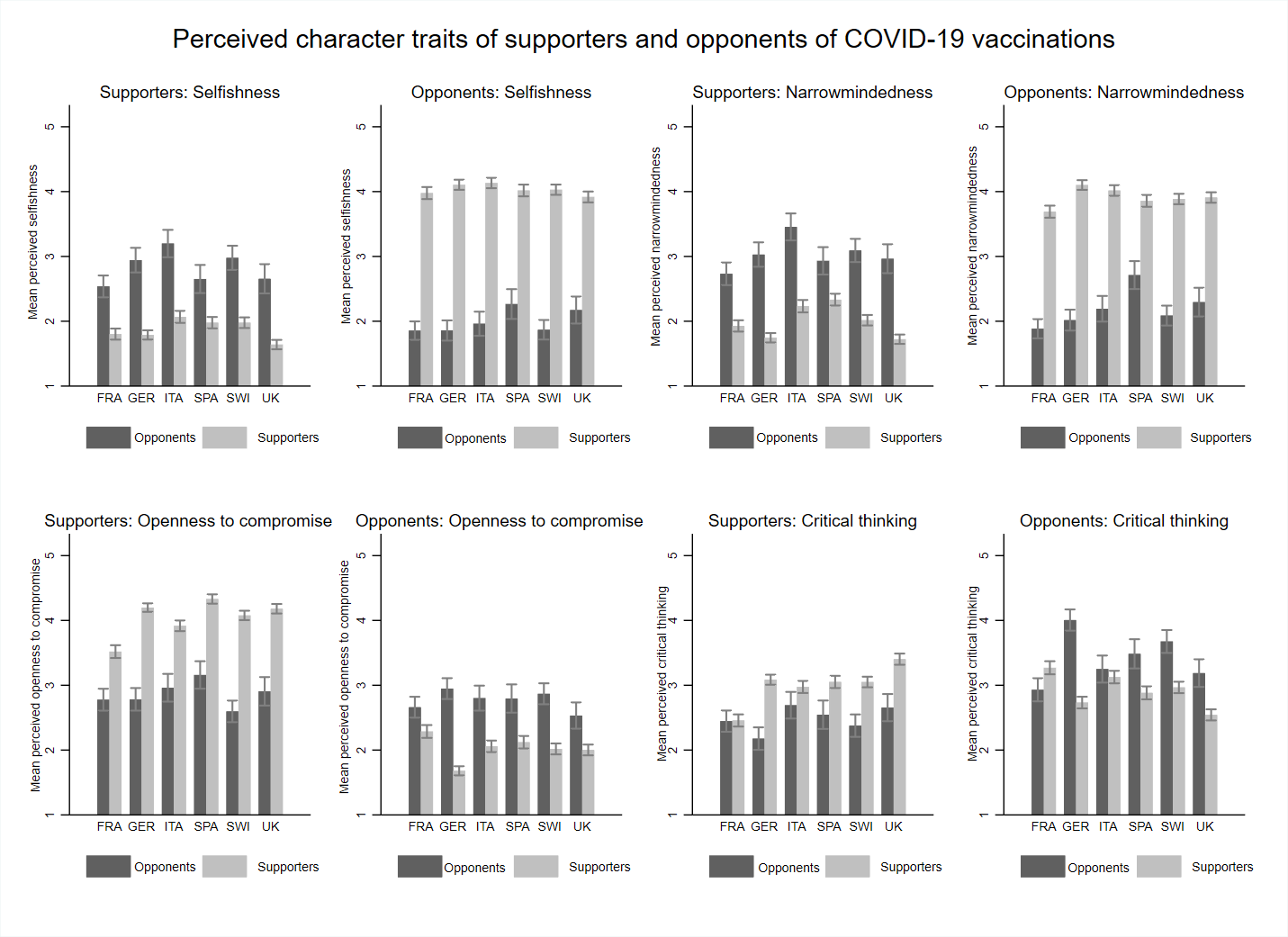


Figure 1 Perceived character traits of COVID-19 vaccination supporters and opponents by group status and country. Notes: Figure shows mean perceived character traits of vaccination supporters and opponents separated by vaccination supporters and opponents and by country with 95% confidence intervals. Reading example for France in the upper left panel: in France vaccination opponents perceive vaccination supporters to be selfish with an average of 2.54 while vaccination supporters perceive vaccination supporters to be selfish with an average of 1.80 on scale from 1 to 5. This difference is significant as can be seen by the non-overlapping of the confidence intervals.

**Supplement E Analyses of potential correlates**

Using linear regression models we evaluate potential correlates of opinion-based affective polarization regarding COVID-19 vaccination. A more detailed description of the variables can be found in the methods section. Figure 2 is a coefficient plot with both measures of affective polarization as dependent variables and sociodemographic, psychological, and ideological correlates across our six countries as independent variables. The first thing to note is that the results are quite similar across countries and measures of affective polarization. Four variables stand out as they have relatively consistent and significant coefficients across all six countries.

First, older respondents are significantly more likely to express affective polarization than younger respondents. For the thermometer measure, the coefficient is relatively strong across the six countries ((β) = 0.09, CI = [0.02, 0.17]), (β)GER = 0.21, CI = [0.15, 0.28], (β)ITA = 0.13, CI = [0.08, 0.18], (β)SPA = 0.07, CI = [0.00, 0.13], (β)SWI = 0.14, CI = [0.08, 0.21], (β) UK = 0.17, CI = [0.09, 0.25]). All coefficients are significant at the 95% level. For the character trait measure, all coefficients are significant at the 95% level except for France: ((β) = 0.06, CI = [-0.02, 0.13]), (β)GER = 0.23, CI = [0.16, 0.30], (β)ITA = 0.09, CI = [0.03, 0.15], (β)SPA = 0.07, CI = [0.00, 0.14], (β)SWI = 0.10, CI = [0.03, 0.17], (β) UK = 0.14, CI = [0.05, 0.22]).

Second, consistent with the descriptive evidence above, pro-vaccination individuals express higher levels of affective polarization in all six countries. For the thermometer measure, the coefficient is strong across the six countries ((β) = 0.51, CI = [0.43, 0.58]), (β)GER = 0.48, CI = [0.40, 0.56], (β)ITA = 0.49, CI = [0.42, 0.57], (β)SPA = 0.50, CI = [0.43, 0.57], (β)SWI = 0.52, CI = [0.44, 0.59], (β) UK = 0.43, CI = [0.34, 0.51]). All coefficients are significant at the 95% level. For the character trait measure, the coefficients are smaller but still substantial (FRA(β) = 0.36, CI = [0.29, 0.44]), (β)GER = 0.28, CI = [0.21, 0.36], (β)ITA = 0.36, CI = [0.29, 0.43], (β)SPA = 0.37, CI = [0.30, 0.44], (β)SWI = 0.27, CI = [0.20, 0.35], (β) UK = 0.34, CI = [0.26, 0.41]). They are also significant at the 95% level. It can be argued that proponents perceive vaccine opponents as more threatening than vice versa. Furthermore, since vaccination has been conceived as a form of social contract, proponents may perceive opponents as having broken this contract, resulting in higher levels of dislike^2,5^.

Third, across the Big Five personality traits, conscientious individuals are more likely to express affective polarization. For the thermometer measure as dependent variable, the coefficients of conscientiousness are (β)FRA = 0.05, CI = [-0.03, 0.12]), (β)GER = 0.11, CI = [0.05, 0.18], (β)ITA = 0.05, CI = [-0.02, 0.12], (β)SPA = 0.06, CI = [-0.01, 0.12], (β)SWI = 0.10, CI = [0.02, 0.17], (β)UK = 0.05, CI = [-0.02, 0.12]). The coefficients are significant in Germany (95% level), Spain (90% level), and Switzerland (95% level). For the character trait measure the coefficients are more consistent and all are significant at the 95% level: (β)FRA = 0.13, CI = [0.06, 0.20]), (β)GER = 0.12, CI = [0.05, 0.20], (β)ITA = 0.10, CI = [0.02, 0.17], (β)SPA = 0.12, CI = [0.05, 0.18], (β)SWI = 0.14, CI = [0.07, 0.22], (β)UK = 0.14, CI = [0.07, 0.21]. Two explanations are logical for these results. Conscientious people are, in general, strongly caught up in their world views and thus are oriented towards the status-quo^38^. Thus, for conscientious individuals who support vaccinations a social contract should be upheld making those that oppose it less sympathetic, while for conscientious individuals who oppose vaccinations newly developed vaccinations and accompanying measures might thus be seen as a threat resulting in affective polarization towards the out-group.


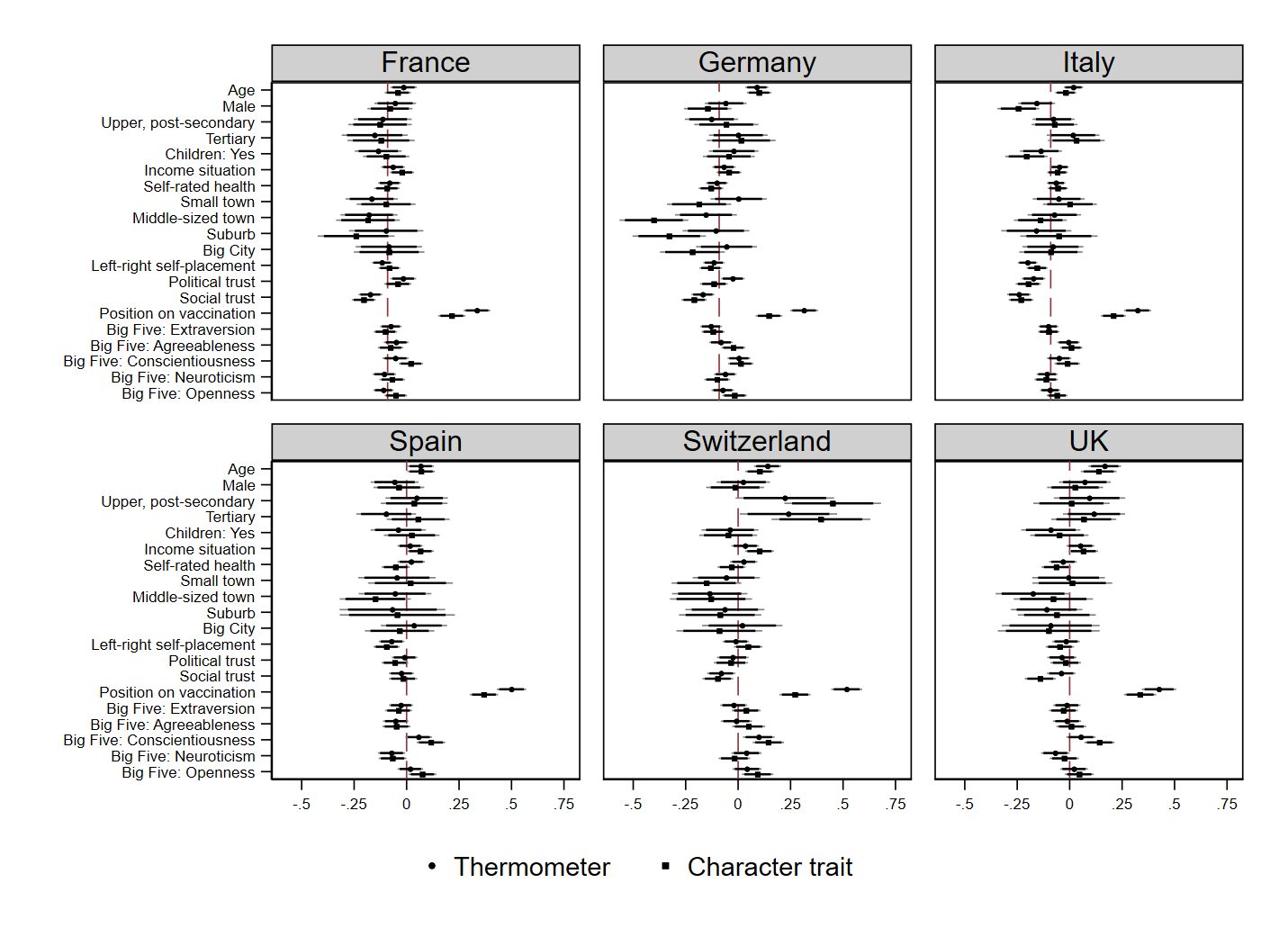


Figure 2 Socio-demographic and psychological correlates of opinion-based affective polarization regarding COVID-19 vaccination. Notes: Linear regression analyses of opinion-based affective polarization regarding COVID-19 vaccination. Figure shows standardized linear regression coefficients with 95% (light gray bars) and 90% (black) confidence intervals. Circles refer to models with thermometer ratings as the dependent variable; squares refer to models with trait ratings as the dependent variable.

Fourth, people with higher levels of generalized trust are less affectively polarized in all countries except Spain and the UK. Using the thermometer measure as the dependent variable, the coefficients of social trust are (β)FRA = -0.10, CI = [-0.16, -0.03]), (β)GER = -0.09, CI = [-0.16, -0.03], (β)ITA = -0.18, CI = [-0.25, -0.11], (β)SPA = -0.03, CI = [-0.09, 0.04], (β)SWI = -0.09, CI = [-0.15, -0.01], (β)UK = -0.04, CI = [-0.11, 0.03]). For the character trait measure the coefficients are (β)FRA = -0.14, CI = [-0.20, -0.07]), (β)GER = -0.14, CI = [-0.21, -0.07], (β)ITA = -0.17 CI = [-0.24, -0.10], (β)SPA = -0.02, CI = [-0.08, 0.05], (β)SWI = -0.10, CI = [-0.17, -0.02], (b)UK = -0.14, CI = [-0.21, -0.07]. Except for Spain all coefficients are significant at the 95% level. People with generalized trust have generally a more inclusive view of society, so they may be less likely to draw sharp group boundaries and thus are less likely to express affective polarization ^39^.

Interestingly, political ideology is not systematically linked to affective polarization regarding the attitudes towards COVID-19 vaccinations. This undergirds the conjecture that opinion-based affective polarization regarding the COVID-19 vaccination is not a pure reflection of left-wing and right-wing ideological positions but follows a distinct logic of social conflict. However, it is important to note that all analyses presented here provide an exploratory picture that does not show causal relationships, nor does it show a definitive set of correlates. We also tested measures of pandemic threat such as fear of infection, economic and social threats as well as the question of whether a respondent was infected with the virus. None of these variables is systematically related to affective polarization. Results are available upon request. We split our sample to distinguish between pro- and anti-vaccination groups, too. The analyses are presented in figures 3 and 4. While the correlations for pro-immunization groups are consistent with the full-sample analyses, there are almost no consistent relationships for anti-immunization groups. However, the number of observations for opponents is generally relatively small (France = 167, Germany=121, Italy=122, Spain=112, Switzerland=146, UK=103), which cautions us against reading too much into the null results.


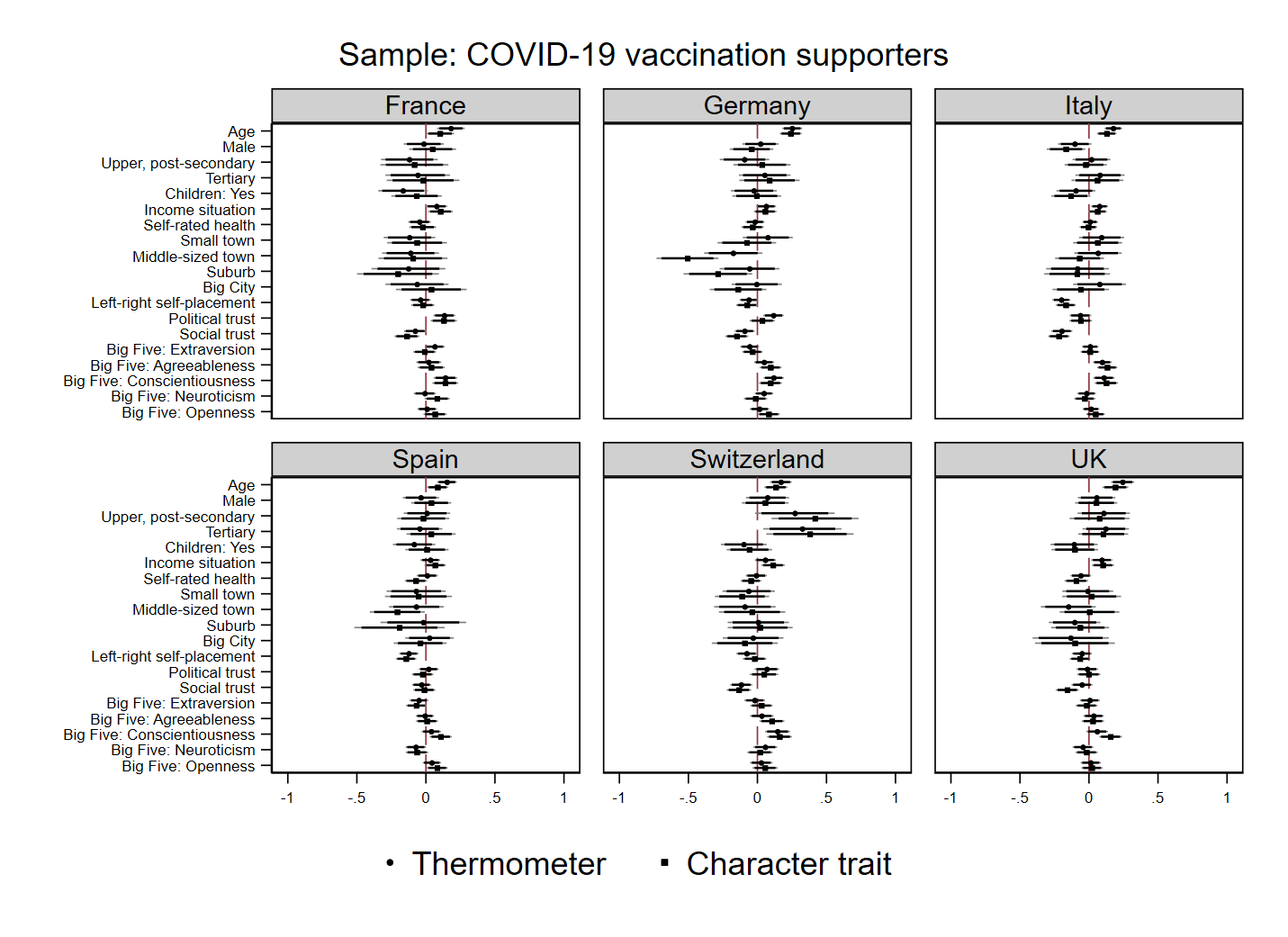


Figure 3 Socio-demographic and psychological correlates of opinion-based affective polarization regarding COVID-19 vaccination for vaccination supporters. Notes: Linear regression analyses of opinion-based affective polarization regarding COVID-19 vaccination. Figure shows standardized linear regression coefficients with 95% (light gray bars), and 90% (black) confidence intervals. The sample is restricted to supporters of the vaccination. Circles refer to models using thermometer ratings as dependent variable; squares refer to models using character trait ratings as dependent variable.


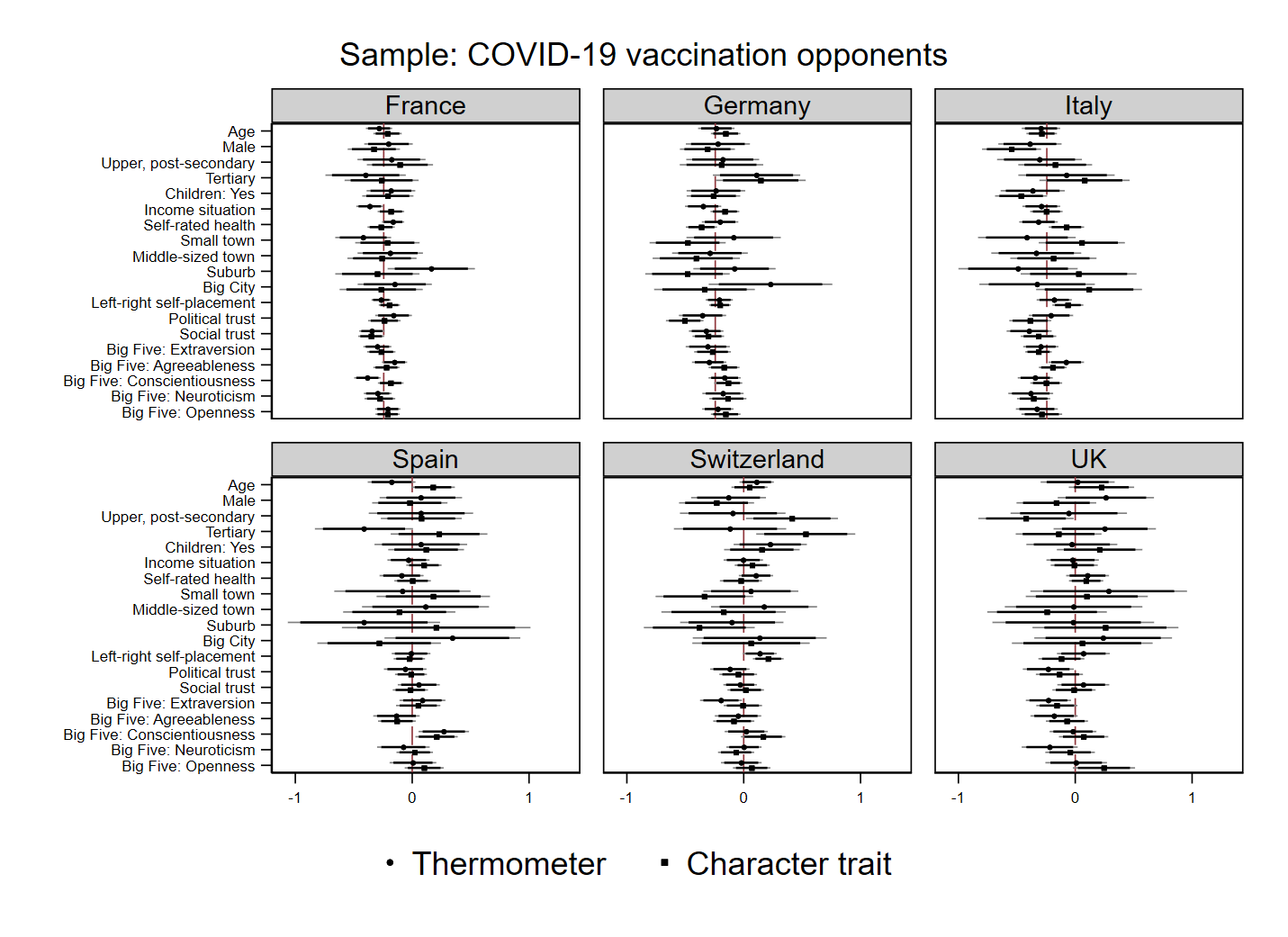


Figure 4 Socio-demographic and psychological correlates of opinion-based affective polarization regarding COVID-19 vaccination for vaccination opponents. Notes: Linear regression analyses of opinion-based affective polarization regarding COVID-19 vaccination. Figure shows standardized linear regression coefficients with 95% (light gray bars), and 90% (black) confidence intervals. The sample is restricted to opponents of the vaccination. Circles refer to models using thermometer ratings as dependent variable; squares refer to models using character trait ratings as dependent variable.
